# Early Transcriptomic Response to OxLDL in Human Retinal Pigment Epithelial Cells

**DOI:** 10.3390/ijms21228818

**Published:** 2020-11-21

**Authors:** Diwa Koirala, Sarka Beranova-Giorgianni, Francesco Giorgianni

**Affiliations:** Department of Pharmaceutical Sciences; The University of Tennessee Health Science Center, Memphis, TN 38163, USA; koiraladiwa@gmail.com

**Keywords:** retinal pigment epithelium, oxidized LDL, transcriptome, oxidative stress, drusen

## Abstract

In the sub-retinal pigment epithelium (sub-RPE) space of the aging macula, deposits of oxidized phospholipids, oxidized derivatives of cholesterol and associated oxidized low-density lipoproteins (OxLDL) are considered contributors to the onset and development of age-related macular degeneration (AMD). We investigated the gene expression response of a human-derived RPE cell line exposed for short periods of time to non-cytotoxic levels of OxLDL or LDL. In our cell model, treatment with OxLDL, but not LDL, generated an early gene expression response which affected more than 400 genes. Gene pathway analysis unveiled gene networks involved in the regulation of various cellular functions, including acute response to oxidative stress via up-regulation of antioxidative gene transcripts controlled by nuclear factor erythroid-2 related factor 2 (NRF2), and up-regulation of aryl hydrocarbon receptor-controlled detoxifying gene transcripts. In contrast, circadian rhythm-controlling genes and genes involved in lipid metabolism were strongly down-regulated. Treatment with low-density lipoprotein (LDL) did not induce the regulation of these pathways. These findings show that RPE cells are able to selectively respond to the oxidized forms of LDL via the up-regulation of gene pathways involved in molecular mechanisms that minimize cellular oxidative damage, and the down-regulation of the expression of genes that regulate the intracellular levels of lipids and lipid derivatives. The effect on genes that control the cellular circadian rhythm suggests that OxLDL might also disrupt the circadian clock-dependent phagocytic activity of the RPE. The data reveal a complex cellular response to a highly heterogeneous oxidative stress-causing agent such as OxLDL commonly present in drusen formations.

## 1. Introduction

Age-related macular degeneration (AMD) is the leading cause of legal blindness in the Western world [1]. The pathogenesis of AMD is not fully understood, with age being the major risk factor, though some genetic determinants and environmental factors are known to play an important role in the progression of the disease [2,3,4,5]. Oxidative stress and inflammation are considered main events leading to the pathophysiology of the disease [6,7]. The retinal pigment epithelium (RPE), the principal tissue affected in early-stage AMD, is considered a major site of oxidative stress in the retina due to its location: between the photoreceptor outer segment, exposed to photo-oxidative light energy, and the choroid with a high oxygen content. In this study, we focused on oxidized low-density lipoprotein (OxLDL), which is a major component of drusen and a known inducer of cellular oxidative stress with pro-inflammatory properties [8]. OxLDL generates from low-density lipoprotein (LDL) in a pro-oxidant tissue environment in which lipids and proteins become oxidatively modified [9]—it comprises a heterogeneous group of macromolecular complexes consisting of various classes of oxidized lipids associated with native or oxidized apolipoprotein B (apoB). As a structurally altered “modified self” form of LDL, OxLDL bears lipid peroxidation-derived structural moieties. These so-called oxidation-specific epitopes (OSEs) enable recognition of OxLDL by cellular and soluble pattern recognition receptors (PRRs) that mediate clearance of OxLDL as part of a protective response by the innate immune system to maintain cellular homeostasis [10]. The transcriptome response of ARPE-19 cells to oxidative stress induced with various agents has previously been reported. Weigel et al. analyzed the transcriptome alteration in ARPE-19 cells after a 4-h exposure to various pro-oxidizing agents including H_2_O_2_, 4-hydroxynonenal and tert-butylhydroperoxide [11]. Cano et al. performed a transcriptomics analysis of ARPE-19 cells exposed for 24 h to cigarette smoke extract (CSE) [12]. Yamada et al. studied the effects of OxLDL-induced oxidative stress in ARPE-19 cells and focused on the cellular response after 48 h of treatment [13].

To obtain a comprehensive outlook of the early transcriptome alterations induced by OxLDL exposure, we performed microarray gene expression analyses in ARPE-19 cells following 2- and 4-h treatments with non-toxic doses of OxLDL. We report that various gene pathways including genes involved in antioxidative defense mechanisms, circadian rhythm regulation and lipid metabolism are modulated in a time-dependent and selective fashion by the OxLDL treatment. Gene expression changes as an early response to OxLDL exposure in RPE cells could help to better understand the early molecular events in the pathogenesis of AMD and provide new opportunities to identify potential druggable targets.

## 2. Results

**Figure A1:**
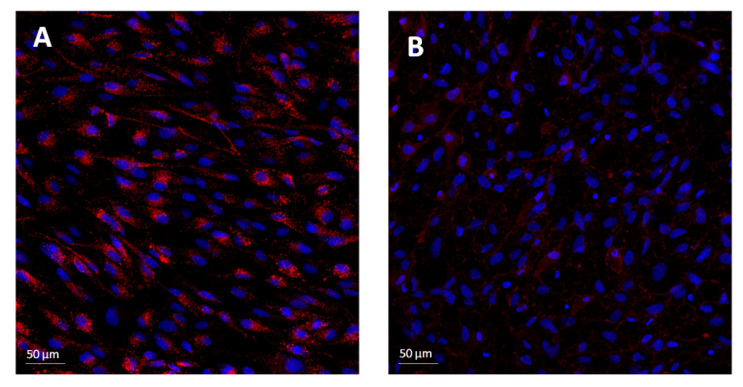
Uptake of OxLDL via CD36. Cells were incubated with fluorescently labeled OxLDL in: (**A**) serum-free medium; (**B**) serum-free medium in the presence of the CD36 inhibitor SSO (Red: fluorescently labeled oxLDL; blue: DAPI nuclear stain, image bar 50 µm).

**Figure A2:**
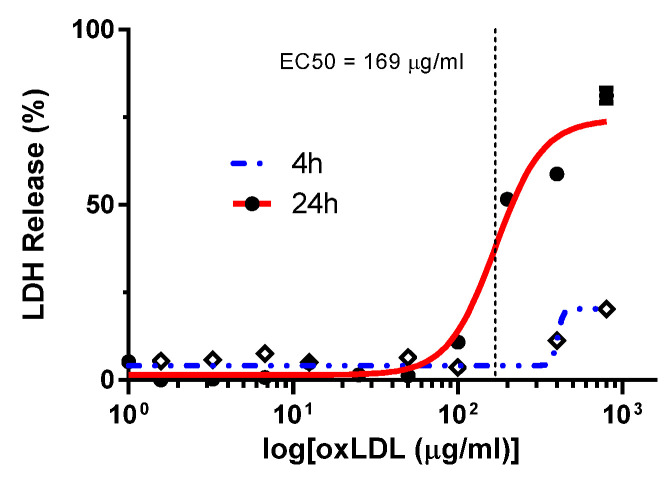
Cytotoxicity profiles of OxLDL and LDL in ARPE-19 cells.

Accumulation of OxLDL is believed to contribute to the pathogenesis of AMD by establishing a low-grade, chronic inflammatory state in the macula. The molecular mechanisms that link the accumulation of OxLDL to the development of AMD are poorly understood. Recent studies in cultured human RPE cells suggest that OxLDL-induced cytotoxicity is mediated by binding through the CD36 cellular scavenger receptor and the activation of the NLRP3 inflammasome [14]. In agreement with this and other studies [15], we confirmed that ARPE-19 cells internalize OxLDL and that CD36 is a major contributor to the cellular uptake of OxLDL. Following pretreatment of the cells with the CD36-specific irreversible inhibitor sulfo-N-succinimidyl oleate (SSO) [16], we observed a drastic decrease in OxLDL uptake as measured by confocal microscopy imaging analysis (please see Appendix A Figure A1) and quantitative flow cytometry (data not shown). To allow for the detection of a robust and early gene expression response without significant cytotoxicity, we tested various exposure times and OxLDL concentrations. We established that 2- and 4-h treatment of ARPE-19 cells with 100 µg/mL of OxLDL had no detectable cytotoxicity as measured by the LDH cytotoxicity assay (Appendix A Figure A2), and this dose was selected for subsequent transcriptomics analyses.

**Figure 1:**
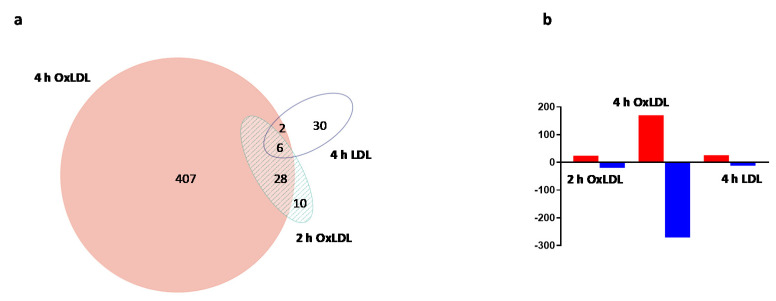
Overall gene expression changes induced by oxidized low-density lipoproteins (OxLDL) or LDL treatment in ARPE-19 cells. (**a**) Total number of treatment-specific and common genes differentially expressed following exposure of the cells to OxLDL (2 and 4 h) and LDL (4 h) (fold change ≥ 1.5, FDR < 5%). (**b**) Number of significantly up- and down-regulated genes following exposure to OxLDL (2 and 4 h) and LDL (4 h) (fold change ≥ 1.5, FDR < 5%).

**Figure 2:**
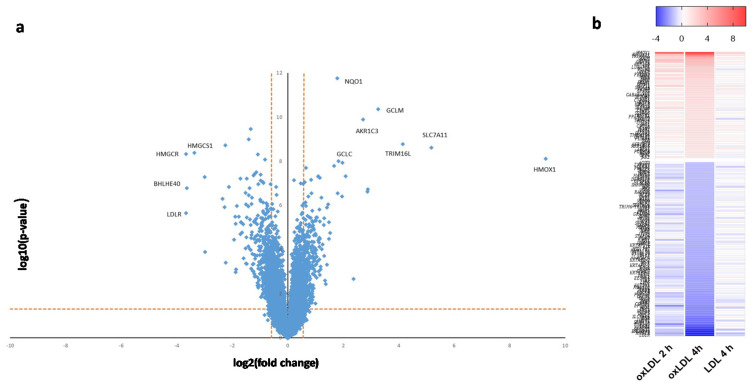
Analysis of differential gene expression in ARPE-19 cells following OxLDL or LDL treatment. (**a**) Scatter plot of the mean expression fold change versus significance following 4-h treatment with OxLDL. The horizontal dashed line represents the threshold limits for statistical significance (q = 0.05); the vertical dashed lines represent fold change threshold limits (≥ ±1.5). Down-regulated genes are on the left; up-regulated genes are on the right. Genes with the highest magnitude of expression change are labeled (see text and Table 1 for details). (**b**) Heat map showing time-dependent gene expression patterns following treatment with OxLDL (2 and 4 h) or LDL (4 h). Results for a subset of high-significance genes are shown. Red color represents up-regulation and blue color represents down-regulation. Intensities of the bars indicate the degree of expression change.

Cell treatment with 100 µg/mL of OxLDL for 2 and 4 h generated a reproducible and robust gene expression response. To determine the specificity of the OxLDL-triggered gene expression changes, cells were also treated for 4 h with 100 µg/mL of LDL, the non-oxidized form of OxLDL. The microarray data showed a marked difference in gene expression profiles between the LDL and OxLDL treatment (Figure 1a; see also Supplementary Table). The 4-h OxLDL treatment caused the highest number of differentially expressed genes: 443 genes were differentially expressed as compared to controls, whereas the LDL treatment caused expression changes in 38 genes (fold change ≥ 1.5, FDR < 5%). Only eight genes were commonly up- or down-regulated between the two treatment groups (Figure 1a). The OxLDL-induced alteration in gene expression was time-dependent, with an increase in the number of differentially expressed transcripts at 4 h (Figure 1a). Interestingly, the data showed that in response to 4-h OxLDL treatment, the highest number of differentially expressed genes were down-regulated (Figure 1b); however, the transcripts with the largest magnitude in expression change were up-regulated genes (Figure 2a). A heat map generated for the differentially expressed genes (fold change ≥ 2.0, FDR < 5%) provides an overall view of the effects of the different treatments (Figure 2b). From this analysis, it is evident that: (i) OxLDL triggered a much stronger transcriptomics response than LDL; and (ii) the OxLDL effects were time-dependent and much more pronounced after the 4-h treatment.

**Figure 3:**
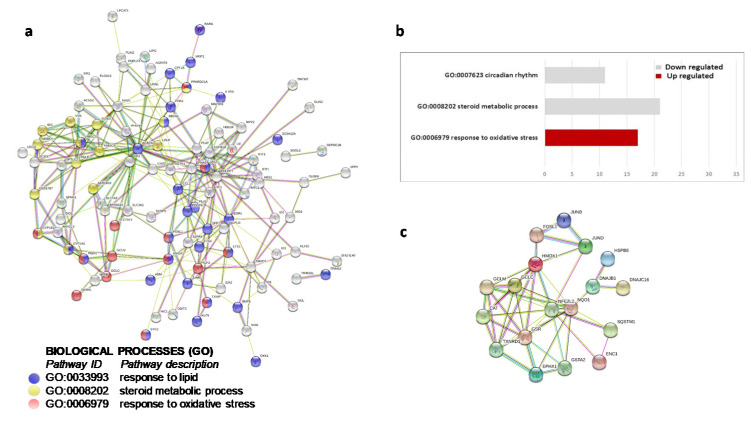
Functional analysis for differentially expressed genes after 4-h OxLDL treatment. Selected functional clustering results by DAVID and STRING. (**a**) Selected functional clustering results by DAVID. Gray bars: biological processes down-regulated by OxLDL; red bars: processes up-regulated by OxLDL. Molecular networks and pathways. (**b**) STRING gene interaction network. Selected, significantly enriched biological processes are highlighted with colored circles. (**c**) STRING gene interaction network encompassing members of NRF2-controlled oxidative stress response.

**Figure 4:**
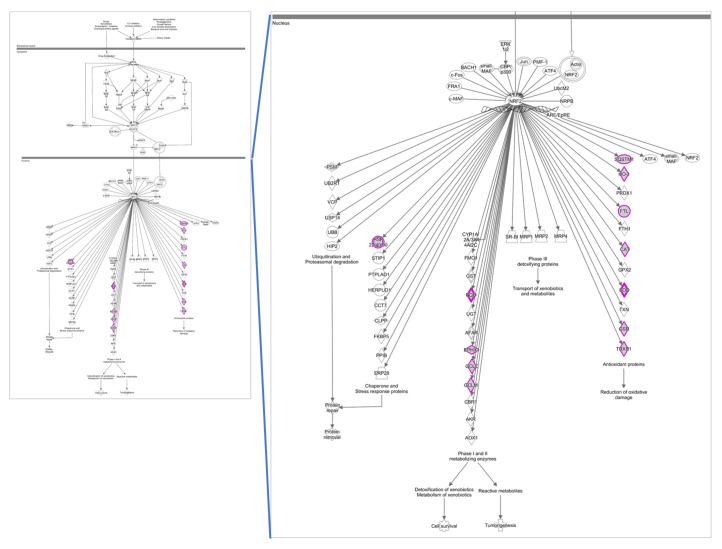
NRF2-controlled pathway up-regulated by OxLDL exposure (4 h). High-significance canonical pathway as revealed by ingenuity pathway analysis; differentially expressed genes from our dataset are indicated with a purple outline.

Due to its higher significance, both in scope and magnitude, we performed functional and pathway analyses on the set of 4-h OxLDL-induced transcripts. These analyses, performed with DAVID and STRING bioinformatics tools, unveiled functional clusters and interaction networks involved in various cellular functions (Figure 3). Among the major biological themes, analysis of the down-regulated transcripts identified functional clusters relevant to lipid biosynthesis and circadian rhythm regulation. Analysis of the up-regulated gene transcripts revealed a group of genes relevant to antioxidative stress response. Ingenuity pathway analysis (IPA) of this group of up-regulated genes identified the nuclear factor erythroid 2–related factor 2 (NRF2) transcription factor as the main regulatory element of the antioxidant response (Figure 4).

Gene expression changes of selected high-significance differentially expressed genes from the identified functional clusters/networks from DAVID and STRING analyses at 4-h OxLDL treatment, and the corresponding changes observed at 2-h OxLDL treatment are shown in Table 1. Gene expression results observed for the 4-h LDL treatment are summarized in Table 2. Taken together, the data shown in Table 1 and Table 2 demonstrate the time dependency and treatment selectivity of the transcriptomic response.

**Figure A3:**
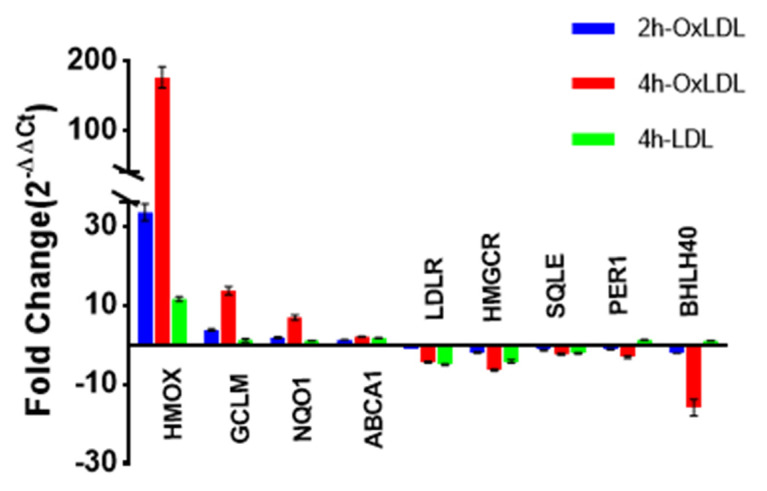
qPCR validation of the expression changes of selected genes following treatment.

Finally, to validate the microarray gene expression results, selected transcripts were quantified by qPCR. The validated transcripts belong to three different functional groups—antioxidative stress response, lipid metabolism and circadian rhythm processes. The qPCR results (Table 3 and Appendix A Figure A3) were overall consistent with the microarray data.

## 3. Discussion

### 3.1. OxLDL and Antioxidative Stress Response

**Figure 5:**
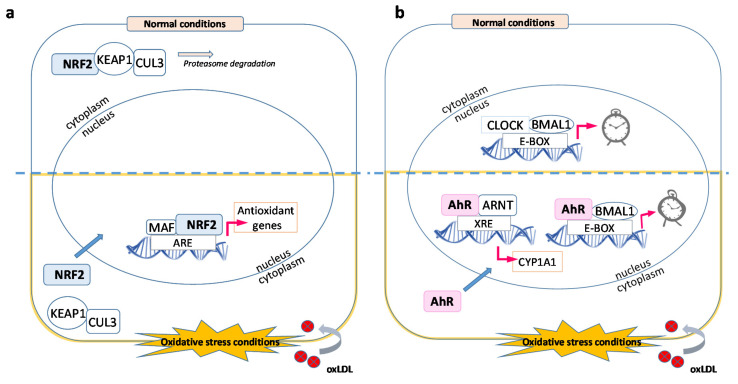
Main gene pathways affected by OxLDL. (**a**) Under normal physiological conditions (top half), NRF2 is part of a multi-protein complex, which is degraded by the proteasome. During OxLDL-mediated oxidative stress (bottom half), this multi-protein complex is disrupted and NRF2 migrates to the nucleus where it activates antioxidant genes by binding to ARE sequences. (**b**) In homeostatic conditions (top half), the CLOCK-BMAL1 complex binds the E-BOX regulatory sequence and activates genes involved in the daily processing of POS. OxLDL accumulation in the RPE cytoplasm (bottom half) promotes the migration of the AhR transcription factor to the nucleus where it promotes the expression of detoxifying enzymes, e.g., CYP1A1. AhR also binds BMAL1, disrupting the CLOCK-BMAL1 complex and thus inhibiting the essential phagocytic and metabolic clearing processes of the retinal pigment epithelium (RPE).

Age-related macular degeneration (AMD) manifests with the formation of drusen, originating from cellular and inflammatory debris that accumulates between the RPE and the Bruch’s membrane. Impairment of the innate immune response, high-fat/cholesterol diet and oxidative stress play a role in the onset and development of AMD. The retina is very vulnerable to oxidative stress: it has a high metabolic rate, high oxygen tension, high content of polyunsaturated fatty acids (PUFAs), presence of retinal pigments and exposure to light. All these factors contribute to the presence of a highly oxidative cellular and tissue milieu in which DNA, lipids and proteins become oxidized. The molecular and cellular mechanisms that lead from oxidative stress to cell dysfunction, cell death and tissue damage are poorly understood. To date, the strongest epidemiological evidence indicates two main risk factors for AMD: (i) genetic polymorphism in genes involved in complement regulation and (ii) environmental factors, i.e., cigarette smoking. Oxidative stress, specifically protein oxidation and its deleterious consequences on cell viability, is sufficient to mechanistically account for both observations. Weismann et al. demonstrated this clearly by showing a mechanistic link between CFH (complement factor H) polymorphism, the highest genetic risk for developing AMD, and oxidative stress-induced protein modifications [17]. To counter the damaging effects of protein oxidation, the RPE has developed numerous antioxidant protective mechanisms. One central component of the protective network is the transcription factor nuclear factor erythroid-2 related factor 2 (NRF2), which induces the expression of various detoxifying enzymes including enzymes of the glutathione redox system, catalases (CAT), superoxide dismutases (SOD) and aldehyde dehydrogenases (ALDH). The NRF2-mediated transcription regulation is a well-characterized antioxidative response mechanism which involves, in the presence of intracellular reactive oxygen species (ROS), the release of the inactive NRF2 protein from its interaction with Kelch-like ECH-associated protein 1 (KEAP1), translocation to the nucleus and dimerization with MAF proteins, binding to the antioxidant response elements (ARE) located in the promoter region of target genes (Figure 5) and initiation of transcription [18]. One of the target genes known to be up-regulated by the activation of NRF2 during the acute phase of an antioxidative response is heme oxygenase 1 (*HMOX1*). Kronke et al. showed that the increase in protein levels of *HMOX1* in human umbilical vein endothelial cells following treatment with oxidized 1-palmitoyl-2-arachidonoyl-sn-glycero-3-phosphorylcholine (OxPAPC), a component of OxLDL, in addition to NRF2 transcription activation, involves the activation of the cAMP-responsive element-binding protein (CREB) through a signaling cascade that includes the mitogen-activated protein kinase p38 and ERK [19]. These findings suggest that cellular antioxidative stress responses to oxidized phospholipids require the activation of multiple signaling cascade pathways that lead to a strong up-regulation of *HMOX1*. In agreement with these studies, functional analyses of our transcriptomics data clustered several of the most up-regulated genes around NRF2 (Figure 3 and Figure 4). Within this cluster of up-regulated transcripts, *HMOX1* had the highest up-regulation at 2- and 4-h OxLDL treatment (>129- and 620-fold increase, respectively; Table 1) in the entire dataset. The *HMOX1* protein (also termed HO-1) catalyzes the breakdown of heme into biliverdin, iron and carbon monoxide [20]. It is up-regulated in chronic and acute inflammation [21,22] and it has antioxidative stress and anti-inflammatory properties [23,24,25,26]. Interestingly, polymorphism of the *HMOX1* gene, which results in an aspartic acid to histidine substitution at position 7 in the protein, has been shown to be associated with increased susceptibility to dry AMD [27]. In addition to *HMOX1*, treatment of ARPE-19 cells with OxLDL also induced a strong NRF2-dependent up-regulation of other gene transcripts whose protein products have antioxidative enzymatic activity including: *GCLM* (glutamate-cysteine ligase modifier subunit), *NQO1* (NAD(P)H dehydrogenase [quinone] 1), *GSR* (glutathione-disulfide reductase), *GCLC* (glutamate-cysteine ligase catalytic subunit), *CAT* (catalase), *TXNRD1* (thioredoxin reductase 1) and *FTL* (ferritin light chain).

### 3.2. OxLDL and Circadian Rhythms

The photoreceptors shed their outer segment (POS) tips daily through cellular processes dependent on light conditions [28]. The RPE plays a critical role in removing the aged POS tips through phagocytosis, a process synchronized with POS shedding and controlled by circadian rhythm mechanisms [29,30]. In fact, there is a strong link between ocular physiology and circadian rhythm with the circadian rhythmicity of the RPE playing a critical role for the support of the photoreceptors and retinal function in all vertebrate animals, including humans [31,32]. Phagocytosis in the RPE is known to increase ROS production, which can alter the renewal process such as delayed termination of shedding or defects in RPE digestion and can cause accumulation of lipofuscin [33]. The accumulation of lipofuscin is one of the major risk factors associated with macular degeneration [34,35]. However, the mechanistic links between phagocytosis of POS, accumulation of ROS and lipofuscin, dysfunction of the circadian rhythm and AMD onset and development are poorly understood [36]. Various studies have shown that disruption of circadian rhythm promotes inflammation in mice as well as in humans [37,38,39], and disruption of circadian rhythmicity in the retina results in increased retinal inflammation in a diabetic mouse model [40]. Moreover, mice treated with dexamethasone and triamcinolone acetonide, powerful anti-inflammatory agents, exhibited up-regulation of genes involved in circadian rhythm regulation one week after treatment [41].

Our studies with OxLDL, a strong pro-inflammatory agent, uncovered a novel regulated gene pathway, which involves gene transcripts known to participate in circadian rhythm mechanisms (Table 1 and Table 3). We identified several circadian rhythm-associated transcripts that were significantly down-regulated in the 4-h treatment group, indicating a time-dependent response. The most down-regulated gene in this group was the class E basic helix-loop-helix protein 40 (*BHLHE40*), a transcription repressor involved in the regulation of the circadian rhythm [42]. Other significantly down-regulated transcripts included the nuclear receptor subfamily 1 group D (*NR1D1*), another transcription repressor that regulates circadian rhythm in a heme-dependent manner [43]. Other down-regulated genes implicated in circadian rhythm regulation were *KLF9* and *KLF10* (kruppel-like factor 9 and 10) [44,45] and *ID1* (DNA-binding protein inhibitor ID1) [46,47]. *KLF9* is a circadian transcription factor that regulates cell proliferation. *KLF10* is a transcriptional repressor that binds to the GC box sequence in the promoter sequence of ARNTL and represses its transcriptional activity. *ID1* regulates the circadian clock by repressing the transcriptional activator activity of the CLOCK/ARNTL heterodimer. Further, as a result of the 4-h OxLDL treatment, we observed a down-regulation (1.8-fold decrease, FDR 0.1) of the period circadian protein homolog 1 transcript (*PER1*), a core component of the circadian clock [48].

The mammalian cellular circadian molecular system consists of core clock genes Period (*PER*) 1 and *PER2*, cryptochrome (*CRY*) 1 and *CRY2*, *CLOCK* and *ARNTL* or *BMAL1* (Aryl hydrocarbon receptor nuclear translocator like) genes [49], and numerous transcription regulators. *CLOCK* and *BMAL1* form the core transactivating components, i.e., the positive components of the feedback loop, and *PER* and *CRY* form the transinhibitory components, i.e., the negative components [50]. These genes generate a circadian rhythm through coupled transcription/translocation feedback loops [41].

Our results suggest a potential role of OxLDL in the inhibition of RPE phagocytic and metabolic clearing processes through interference with circadian rhythm-related molecular processes. How the uptake of OxLDL might interfere with circadian rhythm processes remains to be further investigated. Nevertheless, based on our data and the published literature, we propose a molecular mechanism that could explain the observed down-regulation of circadian rhythm transcripts by OxLDL, which involves the activation of the aryl hydrocarbon receptor (AhR) by oxidized lipid or protein components of OxLDL (Figure 5). Activation of AhR can induce two separate transcription pathways [51]: (i) Activated AhR binds to ARNT and induces xenobiotic metabolism through binding to dioxin response elements (DREs) and transcription of genes with detoxifying functions such as cytochrome P450, family 1, member 1A (*CYP1A1*). Our data show an up-regulation of *CYP1A1.* (ii) Activated AhR binds to BMAL1 and prevents its binding to CLOCK, thus inhibiting the formation of the BMAL-CLOCK heterodimer essential for the transcription of genes needed for POS phagocytosis.

### 3.3. OxLDL and Autophagy

The 4-h OxLDL treatment induced the up-regulation of the autophagy inducer sequestosome1 (*p62/SQSTM1*). The *p62* gene is one of the most important genes involved in regulating the packing and transporting of ubiquitinated, misfolded and aggregated proteins for clearance via autophagy in mammalian cells [52]. Under oxidative stress conditions, induction of the *p62* gene is regulated by NRF2 and contributes to activation of NRF2-controlled target genes through a positive feedback loop that enhances the cellular antioxidative stress response that includes the degradation and removal of the NRF2 inhibitor KEAP1 protein via autophagocytic mechanisms [53]. In RPE cells, it has been shown that *p62* plays a central role in improving cellular viability when the proteasomal pathways are disrupted due to oxidized protein aggregates [54]. Studies in other cell types have shown that *p62/SQSTM1* positively regulates NOD2-induced overexpression of genes related to inflammation such as tumor necrosis factor alpha (TNF-α) and interleukin-1β (IL-1 β) [55]. Our transcriptomics studies indicate that OxLDL induces the p62–KEAP1–NRF2 stress response axis in ARPE-19 cells [53] (Figure 5).

### 3.4. OxLDL and Lipid Metabolism

The accumulation of lipids and lipid derivatives in the sub-RPE space and the association of genes involving lipid metabolism such as *APOE* (apolipoprotein E), *CETP* (cholesteryl ester transfer protein), *LIPC* (hepatic lipase gene) and *LPL* (lipoprotein lipase) with AMD provide support for the postulated role lipids play in the pathogenesis of AMD [56,57,58,59]. In our study, we found a number of differentially expressed genes involved in lipid metabolic pathways, with pronounced expression changes observed after the 4-h OxLDL treatment. This set of highly down-regulated transcripts includes genes involved in fatty acid synthesis, including fatty acid synthase (*FASN*) and acetyl-CoA carboxylase 1 (*ACACA*). Excess intracellular cholesterol is known to suppress the expression of the low-density lipoprotein receptor (*LDLR*) and cholesterol synthesis [60]. In agreement with these findings, our data show that the treatment with OxLDL, and to a much lower level with LDL, had a major impact on the expression of genes whose protein products regulate cholesterol metabolism (Table 1 and Table 2). Following a 4-h treatment with OxLDL, the most down-regulated transcript in the entire dataset was *LDLR*, which showed a 12-fold decrease in expression as compared to the control. LDLR is a transmembrane receptor responsible for the uptake of LDL, but not OxLDL. The down-regulation of the LDLR receptor likely occurs in response to the intracellular release of the OxLDL lipid cargo, which includes cholesterol and various lipid derivatives. The increase in the intracellular levels of cholesterol triggers signaling mechanisms that result in the down-regulation of gene transcripts whose protein products are involved in the uptake of extracellular LDL and the *de novo* synthesis of cholesterol. In addition to a marked down-regulation of LDLR, our data show a significant down-regulation of *INSIG1*, a regulating protein important for the activation of sterol-regulatory element-binding protein (SREBP) transcription factors [61]. Furthermore, our set of down-regulated genes includes a number of enzymes from the *de novo* cholesterol biosynthesis pathway, including 3-hydroxy-3-methylglutaryl-CoA (*HMGCR*)—the rate-limiting enzyme in this pathway—3-hydroxy-3-methylglutaryl-CoA synthase 1 (*HMGCS1*), mevalonate kinase (*MVK*), squalene monooxygenase (*SQLE*) and others (Table 1). In contrast to the strong down-regulation of gene transcripts whose protein products regulate cholesterol uptake and *de novo* synthesis, our data show a significant up-regulation of ATP binding cassette subfamily A member 1 (*ABCA1*), a transporter responsible for efflux of intracellular cholesterol. Taken together, the early response of ARPE-19 to the OxLDL-associated increase in intracellular sterol levels involves attenuation of extracellular cholesterol uptake and of intracellular cholesterol synthesis, together with enhancement of efflux pathways to promote cellular cholesterol release.

## 4. Materials and Methods

### 4.1. Cell Culture and RNA Preparation

The immortalized human retinal pigment epithelium cells ARPE-19 were purchased from ATCC (Manassas, VA, USA) and maintained in DMEM-F12 medium (ATCC, Manassas, VA, USA) containing 2 mM L-glutamine supplemented with 10% fetal bovine serum (ATCC, Manassas, VA, USA) and 100 μg/mL of Primocin antibiotic (Invitrogen, Carlsbad, CA, USA) in a humidified atmosphere with 5% CO_2_ at 37 °C. OxLDL (TBARS: 29–44 nmol MDA/mg) and LDL were obtained from Alfa Aesar (Tewksbury, MA, USA). Cells (150,000) were seeded on 6-well plates and grown until confluent. Serum-starved (24 h) ARPE-19 cells were treated with 100 µg/mL of OxLDL and 100 µg/mL of LDL for 2 and 4 h, respectively. All microarray experiments were performed in multiple biological replicates (*n* = 6). Total RNA was extracted with the Qiagen RNeasy mini kit after DNAse treatment, and RNA quality and quantity were validated with the Agilent Bio Analyzer microfluidics chip RNA Nano 6000. Samples were stored at −80 °C until further use.

For the SSO uptake studies, ARPE-19 cells were grown in coverslips and kept on serum-free medium for 24 h before a one-hour treatment with 100 µM of SSO. After the SSO treatment, the cells were treated with 10 µg/mL of DiI-OxLDL for 5 h. Following removal of excess DiI-OxLDL by consecutive washes with PBS (3 X), the cells were fixed and mounted on glass slides. Cell images were taken with a confocal microscopy (Zeiss 710). Image J software was used to quantify cellular fluorescence.

For the cytotoxicity assay, cells were grown in 96-well plates in serum-containing medium. Before LDL or OxLDL treatment, the cells were grown in serum-free medium for 24 h followed by addition of OxLDL or LDL (0–800 µg/mL). After 24 h of treatment, 50 µL of the medium was transferred to a fresh 96-well flat clear-bottom plate for measurement of lactate dehydrogenase (LDH) release with CytoTox 96^®^ according to the manufacturer’s recommendations. The plate’s absorbance at 490 nm was measured with the Bio Tek Synergy 2 fluorescence reader. The EC50 value was calculated with Prism v. 7.0 (GraphPad). All experiments were performed in multiple biological replicates (*n* = 4).

### 4.2. Microarray Analysis

RNA samples were analyzed with an Affymetrix Human Clariom S array using Affymetrix WT Plus Amplification kit according to the manufacturer’s protocols. Arrays were washed and stained on an Affymetrix Fluidics Station 450 and scanned on an Affymetrix Scanner GCS3000. Data were normalized using the Affymetrix Expression Console. The differentially expressed gene transcripts were determined based on fold change and Benjamini–Hochberg adjusted false discovery rate (FDR). Cutoff values of |fold change| ≥ 1.5 and FDR < 5% were selected.

### 4.3. qPCR

cDNA was prepared from the isolated mRNA (1 µg) by reverse transcription using random hexamers and Maxima H Minus reverse transcriptase according to the manufacturer’s recommendations (ThermoFisher Scientific). Real-time qPCR analyses were performed using gene-specific primers (Table A1) and Power SYBR™ Green PCR Master Mix (ThermoFisher, Waltham, MA, USA) with a StepOnePlus system (ThermoFisher, Waltham, MA, USA). Briefly, the reaction mixture consisted of 10 ng cDNA and 0.6 µM primers in a final volume of 50 µL reaction mixture. Each cycle consisted of a denaturation step at 95 °C for 15 s and annealing and extension steps at 55 °C for 1 min for a total of 40 cycles. A melting curve was generated for each reaction to confirm the absence of non-specific amplification products. GAPDH was used for normalization. All experiments were performed with a minimum of 6 replicates.

### 4.4. Bioinformatics and Statistical Analysis

Microarray data analysis was carried out in Microsoft Excel. The heat map and volcano plot were generated in GraphPad Prism (v. 7.0) and Microsoft Excel, respectively. For functional annotation, transcripts that were significantly up- or down-regulated were analyzed with the DAVID [62], STRING [63] and Ingenuity Pathway Analysis—IPA (QIAGEN Inc., Germantown, TN, USA, https://www.qiagenbioinformatics.com/products/ingenuitypathway-analysis) [64] bioinformatics tools. Since treatment of the ARPE-19 cells by OxLDL for 4 h induced the most pronounced gene expression changes, both in scope and magnitude, the functional analyses focused on the biological alterations at this time point. Additional manual inspection of the functional analysis results and searches of the primary literature were also performed to pinpoint genes that are likely of key mechanistic significance, and to select candidate genes for qPCR validation. Statistical analysis for comparison of differential gene expression between OxLDL- or LDL-treated cells and controls was performed with unpaired Student’s *t*-test in Microsoft Excel.

## 5. Conclusions

In the study reported here, we assessed, at the transcriptome level, the early response of ARPE-19 cells to non-cytotoxic doses of OxLDL. The significant differences observed in the gene transcription response to OxLDL versus the LDL treatment at 4 h highlight the ability of the RPE cells to differentiate between LDL and its oxidized forms bearing oxidation-specific epitopes (OSE). The relatively quick and selective transcriptomics changes, involving various cellular protective pathways, reflect the homeostatic importance these mechanisms have at minimizing the potentially devastating effects caused by the intracellular presence of oxidative stress-inducing species. OxLDL requires transmembrane scavenger receptors for cellular uptake. Depending on the cell type, at least three different scavenger receptors have been described as capable of binding OxLDL: SRAI/II, Lox1 and CD36 [9]. In the RPE, our data and previous published work [14,15] point to CD36 as playing a major role in OxLDL uptake. The mechanisms that link the uptake of OxLDL by CD36 and the subsequent gene expression events are only partially known. In macrophages and platelets, OxLDL binding to the CD36 extracellular domain triggers the interaction of the CD36 C-terminal intracellular domain with fyn and lyn src family kinases, which initiate a series of signaling events that lead to foam cell formation in macrophages and increased reactivity in platelets [65]. In RPE cells, CD36-mediated uptake of OxLDL has been shown to cause activation of the NLRP3 inflammasome and cell death [14]. A recent study has also shown that OxLDL-mediated cytotoxicity involves the activation of TLR-4 (toll-like receptor-4) signaling pathways [66]. These studies confirm the complexity of the cellular pathways involved in RPE cells’ response to OxLDL and the need for additional studies to elucidate such mechanisms. Furthermore, due to the OxLDL heterogeneity reflected in the composition of its active OSE components, multiple cellular response mechanisms are simultaneously activated, including NRF2 and AhR transcription-dependent pathways. At the same time, our data suggest that the non-OSE-containing LDL component of OxLDL induces the expression of genes that mediate the down-regulation of gene pathways involved in lipid metabolism and lipid synthesis. These results depict complex cellular and molecular mechanisms, which involve various cellular response pathways to OxLDL that are only partially overlapping. Ultimately, following the acute response phase, characterized by the activation of the gene expression pathways described in this report, the long-term fate of RPE cells exposed to accumulated OxLDL in the retina depends on the chemical nature and concentration of specific OSEs that represent the active components of OxLDL, on the duration of exposure and on the interaction between RPE and other cell types such as microglial cells. Further understanding of the molecular mechanisms involved and the specific response to selected forms of OxLDL by the RPE will provide valuable knowledge on the etiopathogenesis of AMD.

## Figures and Tables

**Table 1:** Time-dependent expression changes for selected high-significance genes following OxLDL treatment.

Gene	2-h OxLDL/Con ^1,2^	4-h OxLDL/Con
***Response to oxidative stress***		
***HMOX1***	129.6	624.7
***SLC7A11***	6.1	36.0
***TRIM16L***	3.3	17.6
***GCLM***	3.1	9.6
***GCLC***	-	3.5
***NQO1***	1.7	3.4
***AKR1C3***	1.4	6.5
***CYP1A1***	-	2.4
***SQSTM1***	1.4	1.8
***FTL***	1.2	1.8
***CAT***	1.4	1.6
***GSR***	-	1.7
***TXNRD1***	1.2	1.8
***EPHX1***	-	2.8
***Lipid metabolism***		
***ABCA1***	-	2.0
***ACACA***	−1.3	−2.5
***FASN***	−1.4	−2.7
***IDI1***	−1.3	−2.8
***SQLE***	−1.4	−3.2
***INSIG1***	−1.7	−4.7
***HMGCS1***	−1.8	−10.2
***HMGCR***	−2.6	−12.6
***HSD17B7***	−1.4	−2.5
***MVK***	−1.4	−2.6
***SC5D***	−1.4	−2.1
***NSDHL***	−1.4	−2.0
***MVD***	-	−2.0
***LDLR***	−2.0	−12.7
***Circadian rhythm***		
***PPARGC1A***	1.6	2.2
***PHLPP1***	−1.2	−2.0
***NRIP1***	−1.4	−2.2
***KLF9***	−1.4	−2.5
***ETS1***	−1.6	−2.7
***ID1***	-	−2.8
***KLF10***	-	−3.7
***EGR1***	−1.9	−7.9
***PER1***	-	−1.8
***BHLHE40***	−1.8	−12.5
***NR1D1***	-	−1.6

^1^ OxLDL/Con: oxidized LDL/vehicle control. ^2^ Dashes indicate gene expression did not significantly change at the specified time point.

**Table 2:** Changes in gene expression following LDL treatment.

Gene	4-h LDL/Con ^1^
***Response to oxidative stress***	
***HMOX1***	3.2
***GCLM***	1.6
***Lipid metabolism***	
***HMGCS1***	−2.0
***HMGCR***	−2.1
***LDLR***	−1.7
***Other***	
***PORCN***	2.3
***EHD3***	2.0
***NUDT6***	−2.2

^1^ LDL/Con: LDL/vehicle control.

**Table 3:** Validation of expression changes for selected genes by qPCR.

Gene	2-h OxLDL/Con ^1^	4-h OxLDL/Con ^1^	4-h LDL/Con ^1^
***HMOX1***	34.3 ***	175.7 ***	11.9 ***
***GCLM***	4.4 ***	12.6 ***	1.8 **
***NQO1***	2.0 ***	6.8 ***	1.1
***HMGCR***	−2.3 ***	−7.9 ***	−5.1 ***
***ABCA1***	1.3 ***	2.1 ***	1.9 ***
***LDLR***	−1.2 *	−4.7 ***	−5.4 ***
***SQLE***	−1.2 ***	−2.3 ***	−2.1 ***
***BHLHE40***	−2.1 *	−15.9 ***	1.0 *
***PER1***	−1.1 ***	−3.1 ***	1.2

^1^ Values represent GAPDH-adjusted fold changes (2^−ΔΔCt^); * *p* < 0.05; ** *p* < 0.01; *** *p* < 0.001 (*n* = 6).

## Supplementary Materials

Supplementary materials can be found at https://www.mdpi.com/1422-0067/21/22/8818/s1. Supplementary Table: Microarray data sets.

## Abbreviations

RPE	Retinal pigment epithelium
OxLDL	Oxidized low-density lipoprotein
LDL	Low-density lipoprotein
AMD	Age-related macular degeneration
FDR	False discovery rate
NRF2	Nuclear factor erythroid-2 related factor 2
AhR	Aryl hydrocarbon receptor

## Appendix A

**Table A1:** Primers used for qPCR validation of selected transcripts.

Gene	Forward Primer Sequences (5′-3′)
	Reverse Primer Sequence (5′-3′)
***HMOX1***	AGA CTG CGT TCC TGC TCA ACA T
	GGG GCA GAA TCT TGC ACT TTG T
***GCLM***	CAT TTA CAG CCT TAC TGG GAG G
	CGT GCG CTT GAA TGT CAG G
***NQO1***	AAA AGA AGC TGG AAG CCG CA
	AGG ATT TGA ATT CGG GCG TC
***HMGCR***	TCA AAG GGT ACA GAG AAA GCA C
	TAT GCT CCC AGC CAT GGC AG
***ABCA1***	GCA CTG AGG AAG ATG CTG AAA
	AGT TCC TGG AAG GTC TTG TTC AC
***LDLR***	CTG GAC CGG AGC GAG TAC AC
	TGG GTG CTG CAG ATC ATT CTC
***SQLE***	TGG GCA TTG CCA CTT TCA C
	GGC CTG AGA GAA TAT CCG AGA AG
***BHLHE40***	AAT CAT TGC CCT GCA GAG TG
	CGA AGA CTT CAG GTC CCG AG
***PER1***	CTG CTA CAG GCA CGT TCA AG
	CTC AGG GAC CAA GGC TAG TG

## References

[1] Friedman D.S., O’Colmain B.J., Muñoz B., Tomany S.C., Mccarty C., De Jong P.T.V.M., Nemesure B., Mitchell P., Kempen J. (2004). Prevalence of Age-Related Macular Degeneration in the United States. Arch. Ophthalmol..

[2] Nowak J.Z. (2006). Age-related macular degeneration (AMD): Pathogenesis and therapy. Pharmacol. Rep..

[3] Al-Zamil W.M., Yassin S.A. (2017). Recent developments in age-related macular degeneration: A review. Clin. Interv. Aging.

[4] Jang K.-H., Do Y.-J., Son D., Son E., Choi J.-S., Kim E. (2018). AIF-independent parthanatos in the pathogenesis of dry age-related macular degeneration. Cell Death Dis..

[5] DeAngelis M.M., Owen L.A., Morrison M.A., Morgan D.J., Li M., Shakoor A., Vitale A., Iyengar S., Stambolian D., Kim I.K. (2017). Genetics of age-related macular degeneration (AMD). Hum. Mol. Genet..

[6] Rodriguez I.R., Larráyoz I.M. (2010). Cholesterol oxidation in the retina: Implications of 7KCh formation in chronic inflammation and age-related macular degeneration. J. Lipid Res..

[7] Handa J.T., Cano M., Wang L., Datta S., Liu T. (2017). Lipids, oxidized lipids, oxidation-specific epitopes, and Age-related Macular Degeneration. Biochim. et Biophys. Acta Mol. Cell Biol. Lipids.

[8] Ratnayaka J.A., Serpell L.C., Lotery A.J. (2015). Dementia of the eye: The role of amyloid beta in retinal degeneration. Eye.

[9] Levitan I., Volkov S., Subbaiah P.V. (2010). Oxidized LDL: Diversity, Patterns of Recognition, and Pathophysiology. Antioxidants Redox Signal..

[10] Binder C.J., Papac-Milicevic C.J.B.N., Witztum J.L. (2016). Innate sensing of oxidation-specific epitopes in health and disease. Nat. Rev. Immunol..

[11] Weigel A.L., Handa J.T., Hjelmeland L.M. (2002). Microarray analysis of H2O2-, HNE-, or tBH-treated ARPE-19 cells. Free. Radic. Biol. Med..

[12] Cano M., Wang L., Wan J., Barnett B.P., Ebrahimi K., Qian J., Handa J.T. (2014). Oxidative stress induces mitochondrial dysfunction and a protective unfolded protein response in RPE cells. Free. Radic. Biol. Med..

[13] Yamada Y., Tian J., Yang Y., Cutler R.G., Wu T., Telljohann R.S., Mattson M.P., Handa J.T. (2008). Oxidized low density lipoproteins induce a pathologic response by retinal pigmented epithelial cells. J. Neurochem..

[14] Gnanaguru G., Choi A.R., Amarnani D., D’Amore P.A. (2016). Oxidized Lipoprotein Uptake Through the CD36 Receptor Activates the NLRP3 Inflammasome in Human Retinal Pigment Epithelial Cells. Investig. Ophthalmol. Vis. Sci..

[15] Gordiyenko N., Campos M., Lee J.W., Fariss R.N., Sztein J., Rodriguez I.R. (2004). RPE Cells Internalize Low-Density Lipoprotein (LDL) and Oxidized LDL (oxLDL) in Large Quantities In Vitro and In Vivo. Investig. Ophthalmol. Vis. Sci..

[16] Kuda O., Pietka T.A., Demianova Z., Kudova E., Cvacka J., Kopecky J., Abumrad N.A. (2013). Sulfo-N-succinimidyl Oleate (SSO) Inhibits Fatty Acid Uptake and Signaling for Intracellular Calcium via Binding CD36 Lysine 164 SSO ALSO INHIBITS OXIDIZED LOW DENSITY LIPOPROTEIN UPTAKE BY MACROPHAGES. J. Biol. Chem..

[17] Weismann D., Hartvigsen K., Lauer N., Bennett K.L., Scholl H.P.N., Issa P.C., Cano M., Brandstätter H., Tsimikas S., Skerka C. (2011). Complement factor H binds malondialdehyde epitopes and protects from oxidative stress. Nat. Cell Biol..

[18] Tonelli C., Chio I.I.C., Tuveson D.A. (2018). Transcriptional Regulation by Nrf2. Antioxidants Redox Signal..

[19] Krönke G., Bochkov V.N., Huber J., Gruber F., Blüml S., Fürnkranz A., Kadl A., Binder B.R., Leitinger N. (2003). Oxidized Phospholipids Induce Expression of Human Heme Oxygenase-1 Involving Activation of cAMP-responsive Element-binding Protein. J. Biol. Chem..

[20] Maines M.D. (1997). THE HEME OXYGENASE SYSTEM:A Regulator of Second Messenger Gases. Annu. Rev. Pharmacol. Toxicol..

[21] Wang L.J., Lee T.S., Lee F.Y., Pai R.C., Chau L.Y. (1998). Expression of heme oxygenase-1 in atherosclerotic lesions. Am. J. Pathol..

[22] Willis D., Moore A.R., Willoughby D.A. (2000). Heme oxygenase isoform expression in cellular and antibody-mediated models of acute inflammation in the rat. J. Pathol..

[23] Bauer M., Bauer I. (2002). Heme Oxygenase-1: Redox Regulation and Role in the Hepatic Response to Oxidative Stress. Antioxidants Redox Signal..

[24] Bucolo C., Drago F., Maisto R., Romano G.L., D’Agata V., Maugeri G., Giunta S. (2019). Curcumin prevents high glucose damage in retinal pigment epithelial cells through ERK1/2-mediated activation of the Nrf2/HO-1 pathway. J. Cell. Physiol..

[25] Lee T.-S., Chau L.-Y. (2002). Heme oxygenase-1 mediates the anti-inflammatory effect of interleukin-10 in mice. Nat. Med..

[26] Pittalà V., Fidilio A., Lazzara F., Platania C.B.M., Salerno L., Foresti R., Drago F., Bucolo C. (2017). Effects of Novel Nitric Oxide-Releasing Molecules against Oxidative Stress on Retinal Pigmented Epithelial Cells. Oxidative Med. Cell. Longev..

[27] Synowiec E., Szaflik J., Chmielewska M., Wozniak K., Sklodowska A., Waszczyk M., Dorecka M., Blasiak J., Szaflik J.P. (2012). An association between polymorphism of the heme oxygenase-1 and -2 genes and age-related macular degeneration. Mol. Biol. Rep..

[28] Finnemann S.C., Chang Y. (2008). Photoreceptor—RPE Interactions. Visual Transduction and Non-Visual Light Perception.

[29] Lavail M.M. (1976). Rod outer segment disk shedding in rat retina: Relationship to cyclic lighting. Science.

[30] Young R.W., Bok D. (1969). Participation of the retinal pigment epithelium in the rod outer segment renewal process. J. Cell Biol..

[31] Guido M.E., Pico E.G., Contín M.A., Valdez D., Nieto P.S., Verra D.M., Acosta-Rodriguez V.A., De Zavalía N., Rosenstein R.E. (2010). Inner retinal circadian clocks and non-visual photoreceptors: Novel players in the circadian system. Prog. Neurobiol..

[32] Mazzoni F., Safa H., Finnemann S.C. (2014). Understanding photoreceptor outer segment phagocytosis: Use and utility of RPE cells in culture. Exp. Eye Res..

[33] Karan G., Lillo C., Yang Z., Cameron D.J., Locke K.G., Zhao Y., Thirumalaichary S., Li C., Birch D.G., Vollmer-Snarr H.R. (2005). Lipofuscin accumulation, abnormal electrophysiology, and photoreceptor degeneration in mutant ELOVL4 transgenic mice: A model for macular degeneration. Proc. Natl. Acad. Sci. USA.

[34] Dorey C.K., Wu G., Ebenstein D., Garsd A., Weiter J.J. (1989). Cell loss in the aging retina. Relationship to lipofuscin accumulation and macular degeneration. Investig. Ophthalmol. Vis. Sci..

[35] Villegas-Perez M.P. (2005). [Light exposure, lipofuscin and age-related macular degeneration]. Arch. de la Soc. Esp. de Oftalmol..

[36] Fanjul-Moles M.L., López-Riquelme G.O. (2016). Relationship between Oxidative Stress, Circadian Rhythms, and AMD. Oxidative Med. Cell. Longev..

[37] Trammell R.A., Verhulst S., Toth L. (2014). Effects of Sleep Fragmentation on Sleep and Markers of Inflammation in Mice. Comp. Med..

[38] Leproult R., Holmbäck U., Van Cauter E. (2014). Circadian Misalignment Augments Markers of Insulin Resistance and Inflammation, Independently of Sleep Loss. Diabetes.

[39] Maury E., Hong H., Bass J. (2014). Circadian disruption in the pathogenesis of metabolic syndrome. Diabetes Metab..

[40] Wang Q., Bozack S.N., Yan Y., Boulton M.E., Grant M.B., Busik J.V. (2014). Regulation of Retinal Inflammation by Rhythmic Expression of MiR-146a in Diabetic Retina. Investig. Ophthalmol. Vis. Sci..

[41] Smit-McBride Z., Moisseiev E., Modjtahedi S.P., Telander D.G., Hjelmeland L.M., Morse L.S. (2016). Comparison of In Vivo Gene Expression Profiling of RPE/Choroid following Intravitreal Injection of Dexamethasone and Triamcinolone Acetonide. J. Ophthalmol..

[42] Honma S., Kawamoto T., Takagi Y., Fujimoto K., Sato F., Noshiro M., Kato Y., Honma K.-I. (2002). Dec1 and Dec2 are regulators of the mammalian molecular clock. Nat. Cell Biol..

[43] Yin L., Wu N., Curtin J.C., Qatanani M., Szwergold N.R., Reid R.A., Waitt G.M., Parks D.J., Pearce K.H., Wisely G.B. (2007). Rev-erb, a Heme Sensor That Coordinates Metabolic and Circadian Pathways. Science.

[44] Guillaumond F., Grechez-Cassiau A., Subramaniam M., Brangolo S., Peteri-Brunback B., Staels B., Fievet C., Spelsberg T.C., Delaunay F., Teboul M. (2010). Kruppel-like factor KLF10 is a link between the circadian clock and metabolism in liver. Mol. Cell. Biol..

[45] Spörl F., Korge S., Jürchott K., Wunderskirchner M., Schellenberg K., Heins S., Specht A., Stoll C., Klemz R., Maier B. (2012). Kruppel-like factor 9 is a circadian transcription factor in human epidermis that controls proliferation of keratinocytes. Proc. Natl. Acad. Sci. USA.

[46] Duguay D., Cermakian N. (2009). The crosstalk between physiology and circadian clock proteins. Chronobiol. Int..

[47] Ward S.M., Fernando S.J., Hou T.Y., Duffield G.E. (2010). The Transcriptional Repressor ID2 Can Interact with the Canonical Clock Components CLOCK and BMAL1 and Mediate Inhibitory Effects onmPer1Expression. J. Biol. Chem..

[48] Ko G.Y. (2020). Circadian regulation in the retina: From molecules to network. Eur. J. Neurosci..

[49] Ko C.H., Takahashi J.S. (2006). Molecular components of the mammalian circadian clock. Hum. Mol. Genet..

[50] Lowrey P.L., Takahashi J.S. (2011). Genetics of Circadian Rhythms in Mammalian Model Organisms. Genet. Circadian Rhythm..

[51] Jaeger C., Tischkau S.A. (2016). Role of Aryl Hydrocarbon Receptor in Circadian Clock Disruption and Metabolic Dysfunction. Environ. Health Insights.

[52] Dikic I. (2017). Proteasomal and Autophagic Degradation Systems. Annu. Rev. Biochem..

[53] Jain A., Lamark T., Sjøttem E., Larsen K.B., Awuh J.A., Øvervatn A., McMahon M., Hayes J.D., Johansen T. (2010). p62/SQSTM1 is a target gene for transcription factor NRF2 and creates a positive feedback loop by inducing antioxidant response element-driven gene transcription. J. Biol. Chem..

[54] Viiri J., Hyttinen J.M., Ryhanen T., Rilla K., Paimela T., Kuusisto E., Siitonen A., Urtti A., Salminen A., Kaarniranta K. (2010). p62/sequestosome 1 as a regulator of proteasome inhibitor-induced autophagy in human retinal pigment epithelial cells. Mol. Vis..

[55] Park S., Ha S.D., Coleman M., Meshkibaf S., Kim S.O. (2013). p62/SQSTM1 enhances NOD2-mediated signaling and cytokine production through stabilizing NOD2 oligomerization. PLoS ONE.

[56] Neale B.M., Fagerness J., Reynolds R., Sobrin L., Parker M., Raychaudhuri S., Tan P.L., Oh E.C., Merriam J.E., Souied E. (2010). Genome-wide association study of advanced age-related macular degeneration identifies a role of the hepatic lipase gene (LIPC). Proc. Natl. Acad. Sci. USA.

[57] Reynolds R., Rosner B., Seddon J.M. (2010). Serum Lipid Biomarkers and Hepatic Lipase Gene Associations with Age-Related Macular Degeneration. Ophthalmology.

[58] Fritsche L.G., Chen W., Schu M., Yaspan B.L., Yu Y., Thorleifsson G., Zack D.J., Arakawa S., Cipriani V., Ripke S. (2013). Seven new loci associated with age-related macular degeneration. Nat. Genet..

[59] Klein R., Myers C.E., Buitendijk G.H., Rochtchina E., Gao X., de Jong P.T., Sivakumaran T.A., Burlutsky G., McKean-Cowdin R., Hofman A. (2014). Lipids, lipid genes, and incident age-related macular degeneration: The three continent age-related macular degeneration consortium. Am. J. Ophthalmol..

[60] Zhang Y., Ma K.L., Ruan X.Z., Liu B.C. (2016). Dysregulation of the Low-Density Lipoprotein Receptor Pathway Is Involved in Lipid Disorder-Mediated Organ Injury. Int. J. Biol. Sci..

[61] Janowski B.A. (2002). The hypocholesterolemic agent LY295427 up-regulates INSIG-1, identifying the INSIG-1 protein as a mediator of cholesterol homeostasis through SREBP. Proc. Natl. Acad. Sci. USA.

[62] Sherman B.T., Lempicki R.A. (2009). Systematic and integrative analysis of large gene lists using DAVID bioinformatics resources. Nat. Protoc..

[63] Szklarczyk D., Gable A.L., Lyon D., Junge A., Wyder S., Huerta-Cepas J., Simonovic M., Doncheva N.T., Morris J.H., Bork P. (2019). STRING v11: Protein–protein association networks with increased coverage, supporting functional discovery in genome-wide experimental datasets. Nucleic Acids Res..

[64] Krämer A., Green J., Pollard J., Tugendreich S. (2014). Causal analysis approaches in Ingenuity Pathway Analysis. Bioinformatics.

[65] Silverstein R.L., Li W., Park Y.M., Rahaman S.O. (2010). Mechanisms of Cell Signaling by the Scavenger Receptor CD36: Implications in Atherosclerosis and Thrombosis. Trans. Am. Clin. Clim. Assoc..

[66] Yan G., Jiang S., Yu L., Liu S. (2017). Oxidized low density lipoprotein (oxLDL) promotes mitochondrial dysfunction and induces apoptosis in retinal pigmented epithelium cells. Int. J. Clin. Exp. Pathol..

